# Obesity and Adipose-Derived Extracellular Vesicles: Implications for Metabolic Regulation and Disease

**DOI:** 10.3390/biom15020231

**Published:** 2025-02-05

**Authors:** Michele Malaguarnera, Omar Cauli, Andrea Cabrera-Pastor

**Affiliations:** 1 Psychobiology Department, University of Valencia, 46010 Valencia, Spain; michele.malaguarnera@uv.es; 2 Nursing Department, University of Valencia, 46010 Valencia, Spain; 3 Frailty Research Organized Group (FROG), University of Valencia, 46010 Valencia, Spain; 4 Pharmacology Department, University of Valencia, 46010 Valencia, Spain; andrea.cabrera@uv.es; 5 Fundación de Investigación del Hospital Clínico Universitario de Valencia (INCLIVA), 46010 Valencia, Spain

**Keywords:** obesity, extracellular vesicles, adipose-derived extracellular vesicles, type 2 diabetes, cardiovascular system, MAFLD, metabolic syndrome

## Abstract

Obesity, a global epidemic, is a major risk factor for chronic diseases such as type 2 diabetes, cardiovascular disorders, and metabolic syndrome. Adipose tissue, once viewed as a passive fat storage site, is now recognized as an active endocrine organ involved in metabolic regulation and inflammation. In obesity, adipose tissue dysfunction disrupts metabolic balance, leading to insulin resistance and increased production of adipose-derived extracellular vesicles (AdEVs). These vesicles play a key role in intercellular communication and contribute to metabolic dysregulation, affecting organs such as the heart, liver, and brain. AdEVs carry bioactive molecules, including microRNAs, which influence inflammation, insulin sensitivity, and tissue remodeling. In the cardiovascular system, AdEVs can promote atherosclerosis and vascular dysfunction, while those derived from brown adipose tissue offer cardioprotective effects. In type 2 diabetes, AdEVs exacerbate insulin resistance and contribute to complications such as diabetic cardiomyopathy and cognitive decline. Additionally, AdEVs are implicated in metabolic liver diseases, including fatty liver disease, by transferring inflammatory molecules and lipotoxic microRNAs to hepatocytes. These findings highlight the role of AdEVs in obesity-related metabolic disorders and their promise as therapeutic targets for related diseases.

## 1. Introduction

Obesity has become one of the most critical public health challenges of the 21st century, recognized as a global epidemic projected to affect 51% of the world’s population within the next decade [1]. Defined by the excessive accumulation of body fat, obesity is a major risk factor for numerous chronic diseases, including type 2 diabetes, cardiovascular disorders, cognitive impairment, certain cancers, and metabolic syndrome. Metabolic syndrome is characterized by elevated blood glucose levels, reduced high-density lipoprotein cholesterol, increased triglycerides, and high blood pressure [2]. The prevalence of obesity continues to rise at an alarming rate, driven by complex interactions among genetic, environmental, and behavioral factors. In addition to its systemic effects, obesity also induces alterations at the cellular and molecular levels, such as chronic inflammation, insulin resistance, and lipid dysregulation [3,4].

Adipocytes, once considered inert fat storage cells, are now recognized as active regulators of inflammatory balance, metabolic health, and nutrient homeostasis [5,6]. Adipose tissue functions as an endocrine organ, engaging in intricate communication with other organs, including the gut, liver, pancreas, and brain, to regulate processes such as appetite, thermogenesis, and overall body weight [7,8,9]. This communication is closely tied to the inflammatory state of adipose tissue, which plays a pivotal role in obesity-related complications.

In recent years, extracellular particles (EPs), including extracellular vesicles (EVs) and non-vesicular extracellular particles [10,11], have garnered significant attention for their roles in intercellular and inter-organ communication [12], as well as in the regulation of metabolic processes. These nanostructures, released by all cell types, carry bioactive cargoes such as proteins, lipids, and nucleic acids, which can modulate the function of recipient cells [12,13]. In the context of obesity, EVs have emerged as critical mediators of tissue crosstalk, contributing to metabolic dysregulation and the progression of associated diseases [14].

Adipose tissue (AT), categorized into white adipose tissue (WAT) and brown adipose tissue (BAT), plays a central role in energy storage, metabolic regulation, and endocrine signaling [15]. WAT, the predominant form of adipose tissue, functions as a primary energy reservoir and serves as an endocrine organ by secreting adipokines, cytokines, fatty acids, and EPs. It is distributed throughout the body, with major depots in the subcutaneous and visceral regions. In contrast, BAT, which is found in specific regions of the body, is specialized in thermogenesis and energy expenditure, processes stimulated by the sympathetic release of noradrenaline, which activates the β3-adrenergic receptor (β3-AR) on brown adipocytes [16]. BAT’s role in glucose homeostasis and insulin sensitivity further underscores its importance in metabolic health [17,18].

In obesity, WAT undergoes adaptations, including hyperplasia (increased cell proliferation) and hypertrophy (cell enlargement), which contribute to elevated inflammation. This inflammation is driven by immune cell infiltration, particularly macrophages, and is a key factor in the development of insulin resistance and type 2 diabetes [19]. Under negative energy balance, sympathetic release of noradrenaline stimulates lipolysis in WAT, mobilizing free fatty acids to meet the body’s energy demands [20]. On the other hand, BAT has a protective effect on metabolism by promoting energy expenditure and improving insulin sensitivity. BAT is typically reduced in individuals who are obese or elderly and is also associated with cardiovascular health [21,22].

EVs originating from adipocytes are suggested to contribute significantly to the pathophysiology of obesity and its associated comorbid conditions [23]. Recent studies have highlighted the role of adipose-derived EVs (AdEVs), secreted by both white and brown adipocytes, in regulating distant tissues such as the heart, liver or gut. AdEVs, which constitute approximately 80% of circulating EVs in human blood, are a crucial component of the human adipose secretome and carry bioactive molecules that influence the inflammatory state and metabolic function of recipient cells, as observed in both a healthy and pathological heart [24,25]. In obesity, AdEVs have been shown to contribute to gut inflammation by delivering proinflammatory microRNAs (miRNAs) to the intestinal lamina propria, promoting macrophage polarization and aggravating colitis [26]. Furthermore, beige adipocytes, found within WAT but with morphological, functional, and metabolic characteristics of brown adipocytes [27], exhibit thermogenic properties that improve metabolic health and glucose metabolism. Other findings indicate that EVs derived from beige adipocytes can enhance hepatic steatosis and glucose tolerance in diet-induced obese mice [28].

This review explores the intricate relationship between obesity and EVs, focusing on their roles in metabolic regulation, disease pathogenesis and associated-comorbidities. By examining the current evidence, we also aim to highlight the potential of EVs as therapeutic targets for addressing obesity-related disorders such as cardiovascular alterations, type 2 diabetes, and metabolic dysfunction-associated fatty liver disease.

## 2. Influence of EVs in Metabolic Regulation and Obesity-Related Disease

AT is a dynamic organ that adapts its physiological and functional characteristics to regulate metabolic balance during variations in caloric intake. However, chronic or excessive caloric intake can lead to AT dysfunction, characterized by insulin resistance, impaired energy storage, dysregulated lipolysis, and inflammation [29]. Such dysfunction is a key contributor to the onset of metabolic diseases, including type 2 diabetes mellitus, steatohepatitis, and cardiovascular conditions [30,31]. Decades of research underscore the pivotal role of AT health in maintaining overall metabolic stability. The behavior of AT during obesity is partly influenced by dynamic interactions between adipocytes and adipose tissue macrophages (ATMs) [32]. Previous research has identified EVs as a critical mechanism facilitating this communication by serving as carriers of bioactive molecules [33,34,35,36,37]. EVs enable the transfer of molecular signals that regulate inflammation, metabolism, and tissue remodeling, playing a pivotal role in the crosstalk between these cell types and shaping AT function under obese conditions. Studies using high-fat diet-induced or genetically modified rodent models have shown increased AdEV production in obesity [38,39]. Similarly, human studies report elevated circulating EV levels in obese individuals compared to lean subjects, suggesting a role for adipose tissue-derived EVs in these processes [38,40,41]. Altered AdEV profiles have also been observed in obese patients with type 2 diabetes through ex vivo and in vitro studies [40,42].

MicroRNAs (miRNAs) are a class of short noncoding RNAs with a length of about 18–22 nucleotides that are widely produced by all eukaryotic cells [43]. A large number of these miRNAs exist in body fluids [44,45]. Circulating miRNAs are stable in body fluids and can be protected by binding to Argonaute proteins, high-density lipoprotein (HDL), or microRNA protein expression and/or by being encapsulated in EVs [46,47]. The microRNA can control protein expression by binding to mRNA [48,49]. The EVs from diseased sources have been proven to have unique miRNA expression profiles [46]. Recent research indicates that miRNAs are key contributors to the effects of EVs in obesity and metabolic regulation [50,51,52,53]. The miRNA profiles of EVs are influenced by the originating cell type and its physiological state. Importantly, the process of miRNA packaging into EVs is non-random, as specific miRNA sequences are recognized by sorting proteins for selective inclusion [54,55,56]. This regulated secretion of miRNAs via EVs appears to be an evolutionary mechanism for facilitating intercellular and interorgan communication, supporting the maintenance of metabolic homeostasis [38].

### 2.1. Influence of Adipose-Derived EVs on Cardiovascular System

**Figure 1:**
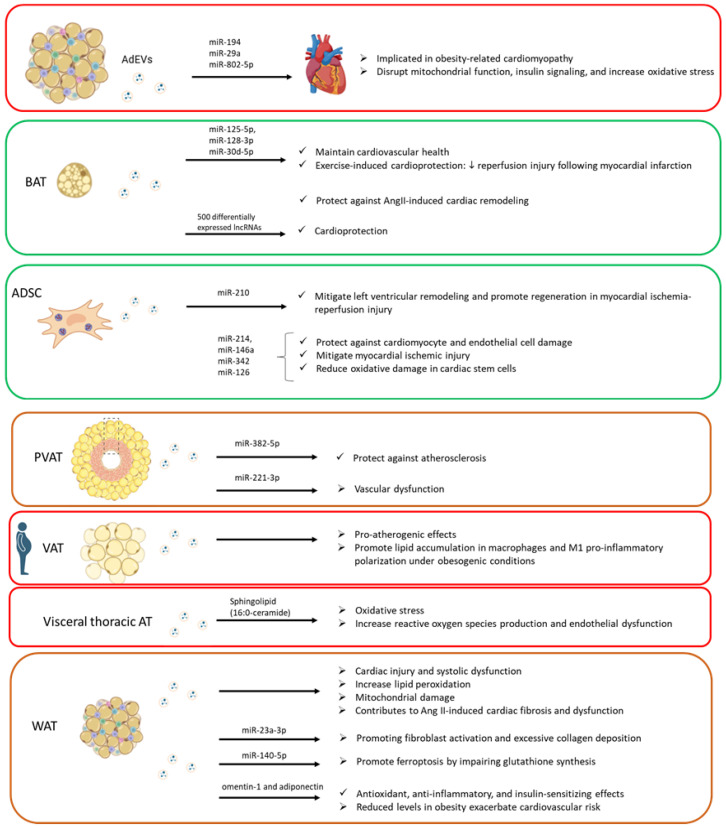
Summary of different types of adipose-derived EVs in the physiology and pathophysiology of the heart (original artwork). AdEVs: adipose-derived extracellular vesicles; AT: adipose tissue; BAT: brown adipose tissue; ADSC: adipose tissue-derived stem cells; PVAT: perivascular adipose tissue; VAT: visceral adipose tissue; WAT: white adipose tissue; Ang II: angiotensin II.

Obesity is a risk factor for cardiovascular diseases. AdEVs appears to play a role in the physiology and pathophysiology of the heart (Figure 1) as they can be taken up by cardiac tissues, as observed many times using the fluorescent labeling of adipose-derived EVs [57,58].

Significant progress has been made in understanding the role of AdEVs secreted by BAT in maintaining cardiovascular health. It has been shown that AdEVs released by BAT play a crucial role in exercise-induced cardioprotection [59]. A 4-week exercise regimen was sufficient to promote BAT expansion, and the resulting EVs effectively reduced reperfusion injury following myocardial infarction. Moreover, comparative RNA sequencing (RNAseq) of circulating EVs and BAT-derived EVs from sedentary and exercised rodents identified miR-125-5p, miR-128-3p, and miR-30d-5p as key mediators of this cardioprotective effect by mitigating apoptosis through inhibition of the MAPK pathway [59]. Other authors also demonstrated that EVs’ communication from BAT to cardiac myocytes and fibroblasts has cardioprotective effects [58]. Their study showed that EVs from β3-AR-activated brown adipocytes protected against AngII-induced cardiac remodeling. The study identified inducible nitric oxide synthase as a key EV cargo contributing to cardiac fibroblast dysfunction and remodeling when BAT was not activated [58]. Additionally, the identification of over 500 differentially expressed lncRNAs in EVs from white and brown adipose tissues may further reveal lncRNAs involved in BAT-mediated cardioprotection in future studies [60]. While brown adipocyte-derived EVs have shown cardioprotective effects, treatment with rosiglitazone, a peroxisome proliferator-activated receptor-gamma (PPARγ) activator used for type II diabetes, has revealed harmful effects. PPARγ is essential for brown adipocyte differentiation [61]. Rosiglitazone induces the release of EVs containing miR-200a, which promotes cardiac hypertrophy by activating mTOR signaling [62]. This mechanism may explain the cardiac hypertrophy and increased heart failure risk observed in preclinical models and clinical trials, leading to restrictions on rosiglitazone use [63].

Other authors demonstrated that EVs derived from adipose tissue-derived stem cells, when transfected with miR-210, mitigated left ventricular remodeling and promoted regeneration in a rat model of myocardial ischemia-reperfusion injury [64]. EVs derived from adipose-derived regenerative cells carrying miR-214, as well as those from adipose-derived mesenchymal stem cells (MSCs) enriched with miR-146a, miR-342, and miR-126, were shown to protect against cardiomyocyte and endothelial cell damage, mitigate myocardial ischemic injury, and reduce oxidative damage in cardiac stem cells, respectively [65,66,67,68].

It has also been shown that EVs derived from perivascular adipose tissue (PVAT) play key roles in regulating vascular physiology and pathology. Several studies reveal that EVs from PVAT protect against atherosclerosis by preventing the formation of macrophages with lipid accumulation through mechanisms such as miR-382-5p, which promotes cholesterol transport and limits lipid accumulation in macrophages [68,69,70]. Interestingly, lower miR-382-5p levels are observed in PVAT-derived EVs from coronary atherosclerosis patients compared to healthy individuals [69]. In contrast, EVs from visceral adipose tissue (VAT) have pro-atherogenic effects, including promoting lipid accumulation in macrophages and M1 pro-inflammatory polarization under obesogenic conditions [71]. Additionally, visceral thoracic adipose tissue has been linked to oxidative stress via EVs secretion containing sphingolipids like C16:0-ceramide, which increases reactive oxygen species production and endothelial dysfunction. Targeting C16:0-ceramide with glucagon-like peptide-1 analog liraglutide shows therapeutic potential, as it reduces plasma levels of this ceramide, which has been associated with increased cardiovascular risk [72]. Other miRNAs in PVAT-derived EVs, such as miR-221-3p, contribute to vascular dysfunction. For instance, under inflammatory conditions, miR-221-3p induces vascular smooth muscle cell phenotypic switching, contributing to early vascular remodeling and dysfunction [73]. However, the exact PVAT cell sources of miR-221-3p in obesity remain unclear.

Obesity drives hyperplastic and hypertrophic remodeling of WAT. This leads to inflammation and long-lasting disruptions in WAT’s endocrine signaling. Notably, WAT-derived EVs are altered in the context of metabolic diseases [41,74], highlighting their potential role in disease progression. Long-term consumption of high-fat diets (HFDs) has been linked to cardiac injury and systolic dysfunction [75,76], with recent evidence implicating EVs derived from adipose tissue macrophages [77]. In obese mice on HFDs, these EVs were associated with increased lipid peroxidation, mitochondrial damage, and an enrichment of miR-140-5p, a microRNA that promotes ferroptosis in cardiomyocytes by impairing glutathione synthesis [77]. Other miRNAs in EVs, such as miR-194, miR-29a, and miR-802-5p, have also been implicated in obesity-related cardiomyopathy, disrupting mitochondrial function, insulin signaling, and increasing oxidative stress [78,79,80]. However, the origins of these EVs—whether from WAT, BAT, macrophages, or hepatocytes—remain unclear. Interestingly, while adipocyte-derived EVs can transport mitochondrial components that impact systemic metabolism [81,82,83], stressed mitochondrial particles in EVs from adipocytes may also activate cardiomyocyte antioxidant defenses, potentially reducing ischemia/reperfusion injury in obesogenic conditions [57]. In obesity, BAT is significantly reduced, leading to the loss of cardioprotective effects [18,21,84]. However, BAT-derived EVs have shown promise; their administration improved cardiac function and metabolic syndrome in mice on HFDs, possibly through direct effects on the heart and enhanced glucose metabolism [85]. Furthermore, EVs from WAT enclose key adipocytokines like omentin-1 and adiponectin, which exert antioxidant, anti-inflammatory, and insulin-sensitizing effects. Omentin-1 was predominantly found in EVs from visceral AT, while high- and medium-molecular-weight adiponectin was present in EVs from subcutaneous AT [86]. Reduced levels of these adipocytokines in obesity likely exacerbate cardiovascular risk [87]. It could be interesting to explore the role of EVs in adipocytokine-mediated protection and their contribution to cardiac damage caused by obesity. The regulatory role of human antigen R, an RNA-binding protein linked to WAT function, may also play a part in EV formation and its association with cardiac fibrosis and inflammation [88,89]. Other authors demonstrated that WAT contributes to angiotensin II (Ang II)-induced cardiac fibrosis and dysfunction through an EV-mediated mechanism [90]. EVs from Ang II-stimulated adipocytes delivered miR-23a-3p to cardiac fibroblasts, promoting their activation and excessive collagen deposition by targeting RAP1, while interventions targeting WAT or miR-23a-3p effectively attenuated these pathological changes [90].

These findings highlight the dual roles of EVs in both mediating cardiac dysfunction in obesity and offering potential therapeutic strategies for mitigating cardiac injury.

### 2.2. Influence of Adipose-Derived EVs on Type 2 Diabetes

EVs play a pivotal role in regulating glucose homeostasis, and their dysregulation in obesity significantly contributes to the onset and progression of type 2 diabetes (T2D).

In obese individuals, AdEVs carry microRNAs, such as miR-155 and miR-29a, which impair insulin signaling by targeting insulin receptor substrates [34,91]. Obesity-induced systemic insulin resistance and chronic inflammation are closely linked, with macrophages playing a critical role in amplifying inflammatory responses though EV-mediated signaling. Recent findings demonstrate that 3T3-L1 adipocytes secrete AdEVs with angiogenic properties, containing approximately 7000 mRNAs and 140 microRNAs [92]. Most adipocyte-specific transcripts and microRNAs were abundant in AdEVs, mirroring donor cell expression. Notably, AdEVs facilitated the transfer of adiponectin and resistin transcripts to RAW264.7 macrophages, while adipocyte-specific genes such as PPARγ2 were detected in serum-derived EVs. Other authors demonstrated that EVs miR-500a-5p derived from adipose tissue macrophages, which is elevated under high-glucose conditions, promoted adipocyte inflammation by suppressing Nrf2 expression [93]. This suppression activated the NLRP3 inflammasome, establishing a connection between macrophage-derived EVs and adipose tissue inflammation [93]. It has also been demonstrated that adipose tissue macrophages release EVs enriched with miR-210-3p, which are delivered to neighboring adipocytes, skeletal muscle cells, and hepatocytes through paracrine and endocrine pathways, thereby affecting insulin sensitivity [94]. Mechanistically, miR-210-3p suppresses glucose transporter type 4 (GLUT4) expression by silencing its mRNA, thereby impairing glucose uptake and reducing insulin sensitivity. Studies have demonstrated that macrophage EVs overexpressing miR-210-3p induce glucose intolerance and insulin resistance in lean mice, while therapeutic inhibition of miR-210-3p in visceral adipose tissue restores glucose tolerance in obese mice, underscoring its potential as a therapeutic target [94]. These findings suggest that targeting adipose tissue macrophage-specific miR-210-3p during obesity could be a promising strategy for managing insulin resistance and T2D.

AdEVs also modulate macrophage polarization through paracrine signaling, with outcomes depending on their miRNA content. For instance, elevated levels of miR-155 in these EVs promote M1 macrophage polarization by targeting the suppressor of the cytokine signaling 1 gene [95]. Conversely, high levels of miR-34a and miR-1224 suppress M2 macrophage polarization by targeting the Krüppel-like factor 4 and Musashi RNA-binding protein 2 (MSI2) genes, respectively [37,96]. This M1 macrophage accumulation leads to the release of pro-inflammatory cytokines in adipose tissue, contributing to insulin resistance.

Other findings highlight the significance of adipocyte EVs as mediators of palmitic acid (PA)-induced metabolic disturbances [97]. Elevated levels of PA, a key factor associated with obesity, have been shown to activate the NF-κB and endoplasmic reticulum (ER) stress pathways in adipocytes, leading to an increased release of miRNAs via EVs. Specifically, obesity-induced increases in PA levels trigger ER stress through NF-κB activation, resulting in elevated levels of EVs miR-4431, miR-548ab/ag, and miR-450a-5p [97]. These miRNAs exacerbate inflammation and metabolic dysfunction, suggesting their utility as biomarkers and therapeutic targets in obesity-related conditions.

In addition, adipose-derived stem cells (ADSC) secrete EVs with anti-inflammatory and metabolic benefits associated with obesity. A study by Zhao, H. et al. [98] showed that ADSC-derived EVs improved metabolic balance in obese mice, enhancing insulin sensitivity by 27.8%, reducing obesity, and alleviating hepatic steatosis. These EVs promoted M2 macrophage polarization, reduced inflammation, and induced the beiging of white adipose tissue (WAT) in diet-induced obese mice. Mechanistically, ADSC-derived EVs activated M2 macrophages through the transfer of active STAT3, which transactivated arginase-1 and fostered anti-inflammatory phenotypes. Furthermore, M2 macrophages increased tyrosine hydroxylase expression and supported ADSC proliferation and lactate production, thereby promoting WAT beiging and restoring metabolic homeostasis in response to high-fat diets [98]. These findings highlight a novel EV-mediated mechanism of ADSC–macrophage interaction that promotes immune and metabolic balance in WAT, offering promising therapeutic avenues for the treatment of obesity and T2D.

A study employing fluorescence AdEV-tracing, SILAC labeling, and (phospho)proteomics investigated the role of AdEVs in glucose regulation [92]. AdEVs were shown to influence glucose regulation by delivering insulinotropic proteins to pancreatic β-cells, where they enhanced first-phase glucose-stimulated insulin secretion (GSIS) through GPCR/cAMP/PKA signaling in murine islets. Interestingly, these insulinotropic effects were specific to AdEVs derived from obese, insulin-resistant mice, reflecting their unique protein cargo and structural characteristics. In vivo, these AdEVs improved insulin secretion and glucose tolerance, highlighting their role in β-cell adaptation to insulin resistance and suggesting their therapeutic potential for improving glucose tolerance [92].

Furthermore, a recent study suggested that phosphotyrosine 1 phosphatase (PTP1B) and protein phosphatase 2 (PP2A), carried by EVs from insulin-resistant individuals, may serve as potential therapeutic targets against insulin resistance in adipose tissue and liver, as well as for preventing the development of obesity [99]. Researchers isolated EVs from individuals with (IR group) and without insulin resistance (n-IR group), and they found that EVs from the IR group were enriched with active PTP1B and PP2A. When these EVs were administered to mice, they impaired systemic, adipose tissue, and liver insulin signaling, and increased adipocyte size and adipogenic gene expression. Inhibition of PTP1B and PP2A activity in IR EVs restored insulin signaling in adipocytes and hepatocytes [99].

Adipocytes are recognized as a major source of EVs miRNA production [100]. miRNA-enriched EVs from adipocytes are not confined to adipose tissue but are also transported to distant insulin-responsive tissues. AdEVs miRNAs influence adipogenesis and adipocyte differentiation through autocrine signaling. For example, miR-122, enriched in adipocyte EVs, promotes adipogenesis by targeting the vitamin D3 receptor gene, which serves as a negative regulator of the sterol regulatory element-binding transcription factor 1 [101]. Research by Ojima et al. [102] revealed that during adipocyte differentiation, EVs enriched with miRNAs support their own differentiation while inhibiting skeletal muscle differentiation.

Furthermore, AdEVs influence gene expression in recipient cells across various organs via endocrine signaling, with effects dependent on the miRNA content and the physiological state of the adipocytes [103,104,105]. For β cells, EVs from healthy adipocytes promote cell survival and insulin secretion, whereas EVs from inflamed adipocytes cause β-cell death and insulin resistance [106]. In skeletal muscle cells, miR-27a-enriched EVs induce insulin resistance by targeting the peroxisome proliferator-activated receptor gamma gene [100]. Animal studies confirmed elevated serum EVs miR-27a levels in obese mice, which were reduced following exercise-induced browning of WAT [107]. Similarly, EVs miR-222 inhibits the insulin receptor substrate 1 (IRS1) gene, impairing glucose uptake in skeletal muscle and hepatocytes, suggesting its potential as a therapeutic target for obesity-induced metabolic syndrome and T2D [108,109].

In hepatocytes, miR-99b-enriched EVs suppress the fibroblast growth factor 21 gene, reducing insulin sensitivity [110]. Conversely, EVs deficient in miR-141-3p impair insulin signaling by upregulating phosphatase and tensin homolog (PTEN), a negative regulator of the PI3K/Akt pathway critical for glucose uptake [111].

It was demonstrated that miR-27a-5p, delivered to pancreatic β-cells via EVs from visceral adipocytes, impaired insulin secretion by downregulating the CaV1.2 calcium channel in β-cells [112]. This led to glucose intolerance in obesity, and blocking miR-27a-5p improved β-cell function and glucose regulation, suggesting it as a therapeutic target for obesity-related type 2 diabetes [112].

Insulin plays a crucial role in regulating miRNA expression and its secretion into EVs from adipocytes [113]. It stimulated the release of miRNAs, such as miR-103-3p and let-7f-5p, which were associated with obesity and disrupted insulin signaling in the liver. The sorting of miRNAs by insulin was driven by specific sequence motifs within the miRNAs and involved RNA-binding proteins that recognized these sequences [113]. This highlights how hormonal control of EV cargo sorting influences inter-organ communication through EVs miRNAs, underscoring its potential as a therapeutic target for conditions like insulin resistance, obesity, type 2 diabetes, and metabolic syndrome (Table 1).

#### Influence of Adipose-Derived EVs on Type 2 Diabetes-Associated Comorbidities

Emerging research has unveiled novel communication mechanisms between adipose tissue and various organs, including the heart and brain, that contribute to the development of comorbidities associated with type 2 diabetes (T2D) and their progression. EVs derived from adipocytes, macrophages, and other adipose cell types can exacerbate systemic metabolic dysfunction and contribute to the development of cardiovascular and cognitive complications. Dysfunctional and senescent white adipocytes have been strongly implicated in the development of T2D and its associated complications [114]. Obesity-induced inflammation and metabolic dysregulation contribute to impaired glucose metabolism and systemic metabolic alterations. Long-term complications induced by diabetes type 2, frequently associated with obesity, have been related to changes in EVs [115] as reported for cardiovascular comorbidities [116,117], diabetic-induced retinopathy [118], or neuropathy [119].

EVs derived from dysfunctional visceral white adipocytes under obesogenic conditions, such as high-fat diets, exacerbate myocardial ischemia/reperfusion (MI/R) injury in diabetic patients compared to healthy individuals [120]. Elevated levels of miR-130b-3p in adipose-derived EVs from diabetic rats and in the plasma of diabetic patients were shown to promote myocardial injury by inducing pro-apoptotic responses in cardiac myocytes [120]. Furthermore, senescent and dysfunctional adipocytes are linked to the development of diabetic cardiomyopathy [114]. A study demonstrated that removal of epididymal visceral WAT alleviated diastolic dysfunction in streptozotocin-induced diabetic mouse models. The findings revealed that EVs from visceral WAT contributed to contractile and mitochondrial dysfunction in isolated cardiac myocytes, with miR-326-3p enrichment in these EVs suppressing Rictor expression and driving cardiac impairments. In addition, miR-802-5p, carried in EVs from hypertrophic adipocytes, has been implicated in cardiac insulin resistance [80]. This microRNA downregulates HSP60, a regulator of insulin-like growth factor-1 receptor (IGF-1R) signaling, which is inversely associated with the progression of diabetic cardiomyopathy [121]. Collectively, these studies emphasize the central role of AdEVs in mediating systemic metabolic dysfunction and cardiac-specific complications in obesity and T2D, while highlighting their potential as therapeutic targets to mitigate disease progression.

EVs also play a dual role in metabolic and cognitive health. In the crosstalk between adipocytes and the brain, a study by Gao et al. [122] showed that EVs secreted by adipocytes from obese mice can be internalized by POMC-like neurons in vitro. When these EVs were transferred to lean mice, they induced increased appetite and body weight gain, likely through the transfer of miRNAs and lncRNAs to POMC neurons, which activated the mTORC1 signaling pathway. Conversely, EVs from adipocytes of lean mice suppressed appetite and attenuated weight gain in mice fed a high-fat diet (HFD) by downregulating mTORC1 signaling. A study revealed that an enzyme involved in NAD+ biosynthesis, the extracellular nicotinamide phosphoribosyltransferase (eNAMPT), is transported via EVs through systemic circulation and significantly declines with age in mice and humans [123]. Increasing circulating eNAMPT levels in aged mice by adipose-specific overexpression of eNAMPT increased NAD+ levels in various tissues, including the hypothalamus, leading to anti-aging effects in female mice. Furthermore, neurons from the hypothalamus were shown to regulate aging and lifespan by promoting adipose eNAMPT release through the sympathetic nervous system [124]. These findings highlight a bidirectional communication between the brain and adipose tissue to sustain NAD+ levels and promote healthy aging.

In the context of obesity, T2D has been linked to cognitive decline [125]. EVs derived from adipose tissue also play a crucial role in cognitive decline associated with T2D. In a recent study, control mice treated with EVs derived from the adipose tissue of HFD-fed mice or diabetic patients displayed hippocampal synaptic loss and cognitive impairments [126]. These effects were attributed to an altered miRNA cargo in the EVs, particularly the enrichment of miR-9-3p, which suppressed hippocampal BDNF expression, a critical factor for synaptic function. Elevated serum levels of EV miR-9-3p have been linked to obesity-related insulin resistance and cognitive decline in diabetes, suggesting that targeting adipose tissue EVs or their miRNA cargo may offer therapeutic strategies for treating cognitive deficits associated with T2DM and insulin resistance [126] (Table 1).

Given their ability to mediate systemic effects on both the heart and brain, adipose-derived EVs represent a critical mechanism through which T2D contributes to the development of comorbidities, such as diabetic cardiomyopathy and cognitive decline. These particles offer potential as therapeutic targets to prevent or mitigate the progression of these complications, providing new avenues for intervention in patients with T2D.

**Table 1:** Effects of cargo in adipose-derived EVs on obesity and type 2 diabetes.

Identified Cargo from AdEVs	Effect on Obesity and T2D	References
miR-155 and miR-29a	Impairs insulin signaling by targeting insulin receptor substrates	[34,91]
140 microRNAs	Angiogenic properties	[92]
Adiponectin and Resistin	Facilitate the transfer of their transcripts to macrophages	[92]
miR-500a-5p	Promotes adipocyte inflammation by suppressing Nrf2 expression	[93]
miR-210-3p	Delivered to neighboring adipocytes, skeletal muscle cells, and hepatocytes, it reduces insulin sensitivity and suppresses glucose transporter type 4 expression	[94]
miR-155	Promotes M1 macrophage polarization by targeting the suppressor of cytokine signaling 1 gene	[95]
miR-34a and miR-1224	Suppress M2 macrophage polarization by targeting the Krüppel-like factor 4 and Musashi RNA-binding protein 2 genes M1 macrophage accumulation leads to the release of pro-inflammatory cytokines in adipose tissue, contributing to insulin resistance	[127,128,129]
miR-4431, miR-548ab/ag, and miR-450a-5p	Exacerbate inflammation and metabolic dysfunction	[97]
STAT3	Anti-inflammatory and metabolic benefits associated with obesity Activates M2 macrophages	[98]
Insulinotropic proteins	Glucose regulation Improve insulin secretion and glucose tolerance, highlighting their role in β-cell adaptation to insulin resistance	[92]
PTP1B and PP2A	Inhibition of PTP1B and PP2A activity in EVs from individuals with insulin resistance restores insulin signaling in adipocytes and hepatocytes	[99]
miR-122	Promotes adipogenesis by targeting the vitamin D3 receptor gene, which serves as a negative regulator of the sterol regulatory element-binding transcription factor 1	[101]
miR-27a	Induces insulin resistance by targeting the peroxisome proliferator-activated receptor gamma gene	[100]
miR-27a-5p	Delivered to pancreatic β-cells, impairs insulin secretion by downregulating the CaV1.2 calcium channel in β-cells. This led to glucose intolerance.	[112]
miR-222	Inhibits the insulin receptor substrate 1 (IRS1) gene, impairing glucose uptake in skeletal muscle and hepatocytes	[108,109]
miR-99b	In hepatocytes, suppress the fibroblast growth factor 21 gene, reducing insulin sensitivity	[110]
miR-141-3p	Deficiency in miR-141-3p impairs insulin signaling by upregulating phosphatase and tensin homolog (PTEN), a negative regulator of the PI3K/Akt pathway, which is critical for glucose uptake	[111]
miR-103-3p and let-7f-5p	Disrupts insulin signaling in the liver	[113]
miR-130b-3p	Promotes myocardial injury	[120]
miR-326-3p	Suppresses Rictor expression and contributes to cardiac impairments	[114]
miR-802-5p	Cardiac insulin resistance Downregulates HSP60, a regulator of insulin-like growth factor-1 receptor (IGF-1R) signaling, which is inversely associated with the progression of diabetic cardiomyopathy	[80]
miR-9-3p	Hippocampal synaptic loss and cognitive impairment Suppresses hippocampal BDNF expression, a critical factor for synaptic function	[126]

A crucial role of the immunological dysregulation leading to systemic inflammation has been suggested as being at the basis of the pathophysiological mechanisms leading to long-term complications in obese patients [130]. EVs’ release from immune cells appear to modulate inflammation and metabolic responses in obesity [3,4] being the macrophages the cells of the immune system those most studied [52,130,131,132]. The macrophages from patients experiencing obesity-associated disorders also release EVs. In atherosclerosis, macrophages stimulated by oxidized low-density lipid (LDL) release EVs that contain regulatory miRNAs or other non-coding RNAs. The adipose tissue macrophages release EVs carrying miR-29a and miR-155 that inhibit activation of adipogenic transcription factor PPAR-γ, resulting in increased insulin resistance and predisposition to type 2 diabetes [130]. Of note, classically activated pro-inflammatory M1 macrophages and adipocytes in the inflammatory environment preferentially release miR-155 [133]. EVs derived from T cells, pro-inflammatory macrophages, B cells, and dendritic cells can release miRNAs that could promote islet β-cell dysfunction and apoptosis [134].

### 2.3. Influence of Adipose-Derived EVs on Liver Lipid Metabolism and Inflammation: Metabolic-Associated Fatty Liver Disease

Hepatic steatosis, defined as fat accumulation in liver cells, represents the initial stage of liver diseases such as non-alcoholic fatty liver disease (NAFLD), metabolic-associated fatty liver disease (MAFLD), and alcoholic liver disease. These conditions differ in their causes and associated comorbidities. NAFLD is diagnosed when hepatic steatosis occurs without significant alcohol intake, secondary causes, or other chronic liver diseases. In contrast, MAFLD, a newer term, emphasizes the metabolic origins of the disease, requiring evidence of hepatic steatosis alongside metabolic dysfunctions, such as overweight/obesity, type 2 diabetes, or metabolic dysregulation [135]. Although the damage in fatty liver disease is multifactorial, a significant contribution arises from fat accumulation in the adipose tissue. Once the adipose tissue’s storage capacity is exceeded, excess lipids are redirected, leading to ectopic fat deposition in the liver [136,137]. This redirection is mediated by free fatty acids released from adipose tissue, which are transported to the liver, bound to albumin, as well as dietary triglycerides delivered via chylomicrons.

AdEVs contribute to MAFLD progression by transferring lipotoxic molecules and inflammatory miRNAs to hepatocytes, further exacerbating metabolic stress [138,139,140]. The inflammatory cargo of these vesicles exacerbates hepatic inflammation and insulin resistance, further driving lipid accumulation and potentially transitioning steatosis to metabolic associated steatohepatitis (MASH) [138].

As highlighted in the previous paragraphs, WAT expands significantly during obesity through hypertrophy and hyperplasia. Human WAT-derived EVs are primarily enriched with cytokines and adipokines, including interleukin (IL)-6, migration inhibitory factor (MIF), MCP-1, adiponectin, resistin, and retinol-binding protein-4 (RBP-4) [141]. These molecules have been shown to inhibit the insulin-induced increase in the pAkt/Akt ratio in HepG2 hepatocytes following treatment with WAT EVs [142].

Other researchers have shown that melatonin can reduce AdEVs containing the adipokine resistin, which helps mitigate hepatic steatosis [143]. AdEVs induced by endoplasmic reticulum stress in adipocytes, collected from mouse models of MASH induced by a HFD or a methionine- and choline-deficient diet, are enriched with Aldo-ketoreductase 1B7, a critical enzyme involved in liver lipid metabolism [144].

Treatment with EVs derived from BAT can partially alleviate HFD-induced hepatic steatosis and reduce serum ALT levels [85]. Using DiR fluorescent dye tracing, Zhou et al. [85] demonstrated that BAT-derived EVs predominantly accumulate in the liver, significantly enhancing hepatocyte oxygen consumption and energy expenditure. Consistent with these findings, proteins in BAT EVs are enriched in mitochondrial components associated with metabolic pathways, likely contributing to their positive effects on liver metabolism [85]. Moreover, it has been demonstrated that EVs derived from BAT significantly improved metabolic syndrome in HFD-fed mice. After intravenous injection, BAT EVs preferentially targeted the liver, reducing the expression of two inflammatory genes (TNFα and IL1β), serum ALT levels, and hepatic lipid accumulation. BAT EVs are capable of delivering functional proteins to the liver, suggesting their potential as a promising therapeutic approach for MAFLD [145]. Additionally, AdEVs have been shown to directly impair hepatic insulin sensitivity through mechanisms involving either miRNAs or proteins. For instance, EVs miR-99b from BAT facilitates communication between AT and the liver by modulating glucose metabolism [110]. It does so by downregulating FGF21 expression in hepatocytes, leading to glucose intolerance and the development of fatty liver [110]. Furthermore, BAT EVs have been shown to promote the browning of WAT, presenting new therapeutic strategies for treating obesity-related metabolic disorders [146]. Additionally, studies indicate that EVs miR-222 derived from WAT is elevated in the livers of obese mice fed an HFD. This miRNA reduces insulin sensitivity in hepatocytes by inhibiting insulin receptor substrate 1 and phospho-AKT, thereby exacerbating hepatic steatosis [147]. Furthermore, EVs miR-155 from ATMs has been found to impair insulin signaling in hepatocytes by targeting PPARγ [34]. Several other miRNAs, such as miR-29, miR-103, miR-223, miR-23a, miR-197, miR-27a, miR-320a, and miR-509-5p, derived from AT EVs have also been linked to metabolic disorders and dyslipidemia through their actions on hepatocytes [148]. Moreover, EVs from obese adipose tissue have been shown to promote liver fibrosis by regulating the expression of fibrosis-associated proteins. This includes the suppression of matrix metalloproteinase-7 in hepatocytes and matrix metalloproteinase-9 in hepatic stellate cells, along with the upregulation of the tissue inhibitor of matrix metalloproteinase-1 and integrin ανβ-5 in both cell types, leading to excessive extracellular matrix production [149]. Interestingly, not all effects of AT EVs are detrimental. EVs enriched with miR-690 from M2 macrophages have been shown to enhance insulin sensitivity in primary hepatocytes when compared to control hepatocytes from obese individuals in vitro [52]. These findings highlight the critical role of AT EVs as regulators of hepatic glucose metabolism and insulin sensitivity.

Endoplasmic reticulum (ER) stress-induced AdEVs have been shown to elevate serum levels of AST, ALT, total cholesterol, triglycerides, and free fatty acids [144]. These AT EVs released under conditions of ER stress also trigger ER stress in the liver, as evidenced by increased expression of markers such as GPR78, CHOP, and IRE1α [144]. Hepatic ER stress is known to exacerbate insulin resistance and promote inflammation through activation of the NF-κB and JNK pathways [150]. In obese mice, the presence of ER stress-induced AT EVs correlates with elevated levels of pro-inflammatory cytokines TNF-α and IL-1β in the liver [144]. Additionally, AT releases EVs containing miR-103, which are internalized by hepatocytes. MiR-103 interacts with PTEN, inhibiting autophagy and contributing to the progression of MASH [151]. These findings highlight the significant impact of ER stress-induced AT EVs on liver dysfunction and inflammation.

AT EVs also play a role in fibrogenesis, the advanced stage of MAFLD. Transforming growth factor beta (TGF-β) is the key pro-fibrogenic growth factor responsible for the activation of hepatic stellate cells into myofibroblast-like cells and for promoting collagen synthesis [152]. Research by Koeck et al. [153] demonstrated that EVs derived from the AT of obese individuals dysregulate the TGF-β signaling pathway in HepG2 cells. This dysregulation is characterized by increased expression of integrin ανβ-5 and tissue inhibitor of matrix metalloproteinase-1 (TIMP-1) and reduced expression of PAI-1 and matrix metalloproteinase-7 (MMP-7). Furthermore, hepatic stellate cells, which are central to liver fibrogenesis, exhibited elevated Smad-3 expression, along with upregulation of TIMP-1, TIMP-4, MMP-9, and integrins [153]. Interestingly, among the MMPs and TIMPs studied, mRNA levels for MMP-2, MMP-3, MMP-12, MMP-14, MMP-19, and TIMP-1 are strongly induced in obese adipose tissues compared with lean tissues [154]. EVs derived from macrophages carry bioactive MMP-14, which has gelatinolytic and collagenolytic activities, thus influencing the development of adipose tissue [155,156]. Other proteins with MMP activity have been implicated in obesity, such as ADAM 10/17. A recent work reported that adipose tissue-selective ablation of ADAM10 results in divergent metabolic phenotypes following long-term dietary manipulation [156], and knocking out ADAM17 in mice leads to extremely lean animals [157]. Interestingly, ADAM10/17 have also been found to be released with EVs [157], which can contribute to adipocytes differentiation and growth of adipose tissue. Another study by Gu et al. [144] linked an increased release of AdEVs under ER stress conditions to higher liver expression of TGF-β1, collagen 4α1, and collagen 1α2. These findings underscore the role of AT EVs in promoting liver fibrosis through multiple molecular mechanisms.

Understanding the molecular composition of these EVs and their liver-targeting mechanisms offers promising avenues for therapeutic intervention to mitigate obesity-induced liver disease. Recently, it has been shown that lifestyle interventions can influence EVs’ profiles, potentially mediating metabolic improvements. A study analyzed 18 Latino adolescents with obesity and hepatic steatosis who underwent a six-month intervention, and results showed a 23% reduction in hepatic fat fraction and smaller EV sizes post-intervention [158]. Moreover, proteomic profiling identified 462 EV proteins, with 113 significantly altered post-intervention, enriched in complementary cascade pathways. Hepatocyte-specific analysis revealed 40 proteins with suggestive changes [158]. These findings suggest EV-derived proteins may contribute to hepatic fat reduction through complement-related mechanisms.

## 3. Conclusions

AdEVs play a central role in the pathogenesis of metabolic diseases, particularly in obesity and its associated complications, such as T2D, cardiovascular dysfunction, and liver diseases. In obesity, the dysregulation of AT metabolism leads to an overproduction of AdEVs, which facilitate communication between adipocytes and macrophages, modulating inflammatory responses, metabolism, and tissue remodeling. These vesicles, enriched with specific microRNAs, regulate glucose homeostasis, insulin sensitivity, and lipid metabolism, contributing to the onset and progression of insulin resistance, hepatic steatosis, and systemic metabolic dysfunction.

The impact of AdEVs extends beyond metabolic regulation to cardiovascular health, where they can either promote or protect against cardiac dysfunction. EVs derived from BAT show cardioprotective properties, reducing myocardial injury and promoting exercise-induced cardioprotection. In contrast, EVs from WAT and perivascular adipose tissue may exacerbate cardiovascular disease by promoting inflammation and vascular remodeling. However, AdEVs from ADSCs have demonstrated therapeutic potential, improving cardiac regeneration and mitigating myocardial injury, highlighting their promise in treating obesity-related cardiovascular conditions.

In liver diseases, AdEVs contribute to the progression of MAFLD by transferring inflammatory molecules and lipotoxic miRNAs to hepatocytes, exacerbating hepatic inflammation, insulin resistance, and lipid accumulation. These processes drive the progression from simple steatosis to more severe forms of liver disease, such as steatohepatitis and fibrosis. AdEVs also play a role in liver fibrosis by dysregulating key pathways, such as TGF-β signaling, and activating hepatic stellate cells, further promoting excessive extracellular matrix production.

Moreover, AdEVs contribute to the systemic effects of obesity-related diseases, influencing organs such as the brain, liver, and heart. In diabetic conditions, the altered miRNA cargo of AdEVs exacerbates insulin resistance and organ-specific damage, including diabetic cardiomyopathy and cognitive decline. These findings underscore the multifaceted roles of AdEVs in mediating inflammation, metabolic dysfunction, and tissue-specific damage.

Importantly, lifestyle interventions, including dietary changes, can influence the profile of AdEVs, potentially mitigating metabolic dysfunction and improving clinical outcomes in individuals with obesity-related conditions. This suggests that targeting AdEVs and their miRNA cargo could offer novel therapeutic strategies to address obesity, type 2 diabetes, cardiovascular diseases, and liver diseases, marking a promising area for future research and clinical application.

## Data Availability

Not applicable.
